# A Functional NIRS Study of Brain Functional Networks Induced by Social Time Coordination

**DOI:** 10.3390/brainsci9020043

**Published:** 2019-02-15

**Authors:** Megumi Mizuno, Tomoyuki Hiroyasu, Satoru Hiwa

**Affiliations:** 1 Graduate School of Life and Medical Sciences, Doshisha University, 1-3 Tatara Miyakodani, Kyotanabe, Kyoto 610-0394, Japan; mmizuno@mis.doshisha.ac.jp; 2 Faculty of Life and Medical Sciences, Doshisha University, 1-3 Tatara Miyakodani, Kyotanabe, Kyoto 610-0394, Japan; tomo@is.doshisha.ac.jp

**Keywords:** functional near infrared spectroscopy, synchronized tapping task, graph theories, mirror neuron, temporal expectation

## Abstract

The ability to coordinate one’s behavior with the others’ behavior is essential to achieve a joint action in daily life. In this paper, the brain activity during synchronized tapping task was measured using functional near infrared spectroscopy (fNIRS) to investigate the relationship between time coordination and brain function. Furthermore, using brain functional network analysis based on graph theory, we examined important brain regions and network structures that serve as the hub when performing the synchronized tapping task. Using the data clustering method, two types of brain function networks were extracted and associated with time coordination, suggesting that they were involved in expectation and imitation behaviors.

## 1. Introduction

Joint action are social interactions in which more than two people coordinate their behavior to change the environment. In daily life, the ability to coordinate one’s behavior with the others’ behavior is essential to achieve a joint action, which is one of the most important components of social interaction [1,2,3]. Neural mechanisms for temporal coordination with external stimuli have been studied using finger-tapping tasks [4]. It reveals that we tend to respond predictively, and not imitatively, to stimuli whose intervals are short and periodic.

It has also been reported that temporal synchronization with periodic stimuli is associated with brain activation in the primary sensorimotor area, ipsilateral cerebellum, premotor cortex, supplementary motor area, and superior temporal gyrus [5,6]. Many studies have focused on the brain activity in responding to periodic or random stimuli; however, the external stimuli in the case of a joint action in daily life is neither periodic nor completely random [7,8]. The joint action can be achieved by temporal coordination between interacting partners, and also by predicting or imitating each other’s behavior. The rhythm of the partner’s behavior can be predicted on the basis of the partner’s intention, strategy, character, and so on. The predictive behavior forms a key component of social interaction.

As one of the simulation models considered herein, for the analysis of the joint action, we focused on the neural basis of temporal coordination with the external stimulus whose interval was non-periodic but predictive. According to our hypothesis, there are two strategies for this coordination: (1) an active strategy by responding prior to the stimulus occurrence; and (2) a passive strategy by following up the stimulus occurrence. Therefore, we aimed to detect the brain activity patterns associated with these two strategies.

The brain activity during a response to the auditory cues was measured using functional near infrared spectroscopy (fNIRS), which quantifies the changes in hemoglobin (Hb) concentration [9]. Although functional magnetic resonance imaging (fMRI), which works on the principle of magnetism, is traditionally used for measuring brain activity, the method has several disadvantages, such as the equipment is noisy and it adds many constraints to the experimental environment. On the other hand, fNIRS can measure brain activity in a condition closer to real life, and it is suitable for experiments involving the use of sound as the equipment noise is less intense than that of fMRI. Moreover, fNIRS is superior in temporal resolution, and we think that it is more suitable for measuring the brain activity for dynamically changing behavior. Furthermore, clinical studies and research on infants employ fNIRS because it is less restrictive for subjects and is highly safe, and, thus, fNIRS has also attracted the attention of the social brain researchers [10].

Furthermore, functional connectivity, which is defined as a statistical dependence between distant neurophysiological activities, has been studied recently. In particular, *connectomics*, in which statistical correlations between brain regions are analyzed as a brain network, has become an active field of research [11,12,13]. Rubinov et al. [14] proposed a method to analyze the features of a functional-network structure based on the graph theory, and revealed the network structures unique to specific diseases by analyzing specific cognitive states using the metrics of graph theory.

In this study, the brain activity patterns were investigated in terms of the brain functional-network structure using graph theoretical analysis.

## 2. Materials and Methods

### 2.1. Participants

Twenty healthy subjects (aged 22 ± 0.6 years, 10 females, all right handed) participated in this experiment after giving written informed consent. This study was carried out in accordance with the research ethics committee of Doshisha University, Kyoto, Japan (approval code: 15098). The room temperature and humidity were controlled during the experiment (21.8 ± 1.0°C, and 78.8 ± 3.6%). Participants performed a synchronized tapping task, and we measured their brain activities during the task using fNIRS.

### 2.2. Behavioral Data Acquisition

#### The Synchronized Tapping Task

**Figure 1:**
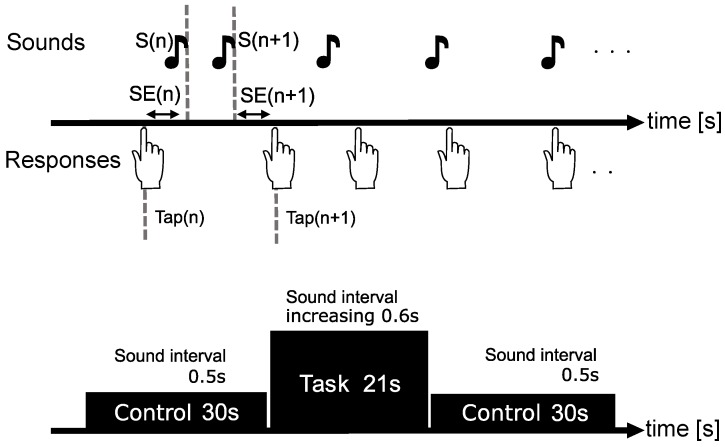
A block design consisting of two controls and one task block. A synchronous tapping task was used, and the subjects were required to respond to the sound stimulus presented at regular time intervals. In the control phase, the sound interval was 0.5 s (S(n+1) − S(n) = 0.5 s), while, in the task phase, the sound intervals were regularly increased (S(n+2) = S(n+1) − S(n) + 0.6 ± 0.03 s).

The synchronized tapping task involved synchronization of tapping with a sound stimulus. Figure 1 shows the experimental design. The experiment consisted of pre-control, task, and post-control phases. The pre-scan time of the fNIRS measurement for the baseline correction was set to 20 s before pre-control. During the control block, the sound was presented at 0.5 s intervals for 30 s. For the task block, sound was presented for 21 s with increasing time intervals of 0.6 ± 0.03 s. Sound stimulus was presented seven times. The subjects were instructed to watch the fixation point on the screen and to press the button in synchrony with the sound presented during both the control and the task block. Sound was presented through an earphone (ATH–ANC 23 Audio Technica Corporation, Tokyo, Japan) and a sine wave of 500 Hz lasting 0.1 s was used. The experiment was implemented with Presentation software (Neurobehavioral system Inc., Albany, NS, Canada). We recorded the subject’s response using a keyboard, and the difference between their response time and stimulus presentation time was used as an indicator of their performances.

### 2.3. fNIRS Data Acquisition

**Figure 2:**
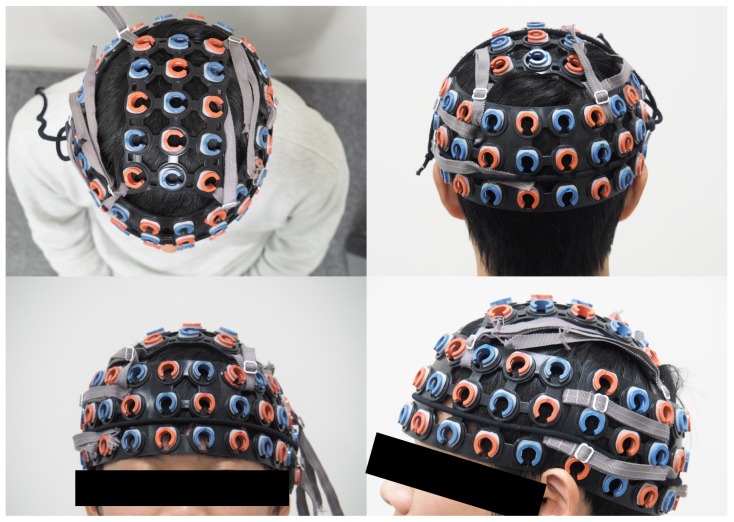
Probe set configuration. Red circles indicate emitters and blue circles indicate detectors. A 3 × 10 probe set (47 channels) was attached to each of the forehead and occipital regions, and a 3 × 5 probe set (22 channels) was attached to the top head region. In total, 116 channels were measured for the whole brain.

The subjects sat in front of a personal computer and performed the synchronized tapping task. The oxy- and the deoxy-Hb concentrations were measured using ETG-7100 fNIRS system (Hitachi, Ltd., Tokyo, Japan), with a sampling frequency of 10 Hz. An fNIRS system consists of irradiation probes and detection probes that are placed on the human head. Near-infrared signals are injected from the irradiation probes into the brain surface, where they diffuse into the cerebral tissue up to a depth of 20–30 mm. Two wavelengths of near-infrared light (695 and 830 nm) were used, and the light reflected by the oxy- or the deoxy-Hb could be distinguished by detection probes. The relative changes in the oxy- and deoxy-Hb concentrations were calculated on the basis of the modified Beer–Lambert law [15]. Figure 2 shows the locations of the fNIRS measurement probes. Two sets of 3 × 10 probes (15 emitters and 15 detecters, forming 47 measurement channels) were attached to the forehead and occipital regions, respectively, according to the reference point of the International 10–20 system. Besides, a 3 × 5 probe set (8 emitters and 7 detecters, forming 22 channels) was attached to the top head region. In total, 116 channels covering the whole brain were set. Each inter-probe distance was fixed at 30 mm. We used a 3D magnetic digitizer stylus (PATRIOT, Polhemus, Colchester, VT, USA) to obtain the relative locations of 10–20 standard positions and fNIRS probes in a real-world coordinate system.

### 2.4. fNIRS Data Processing

#### 2.4.1. Preprocessing

The obtained fNIRS data were band-pass filtered (pass-band: 0.010 Hz to 0.33 Hz) [16,17]. Oxy-Hb data changed per unit time. A step where the change exceeded 0.1 mM·mm was regarded as a motion artifact [18], and the channel data that included such values were excluded from the analysis. Moreover, spatial registration of fNIRS channel location to Montreal Neurological Institute (MNI) space was performed using probabilistic registration and virtual registration toolboxes (available at http://www.jichi.ac.jp/brainlab/tools.html) on the platform for optical topography analysis tools (POTATo) developed by Hitachi, Ltd.

#### 2.4.2. Functional Connectivity Analysis

**Figure 3:**
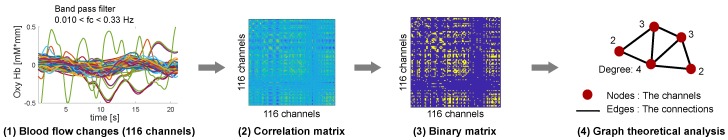
Procedure for the brain function network analysis. (1) The noise associated with Oxy-Hb changes (116 channels) was removed by pre-processing. (2) Pearson correlation matrix of Oxy-Hb concentration changes was calculated. (3) The correlation matrix was binarized by threshold (edge density of 15%). (4) Network feature amount (degree) was calculated based on graph theory. The red dots indicate nodes and the lines indicate edges. The numerical values indicate the number of nodes connected by other nodes.

Figure 3 shows the procedure for fNIRS data processing. To investigate the functional connectivity of the network during the synchronized tapping task, Pearson correlation coefficients for the 116 channels of Oxy-Hb time course during the task block were calculated; we performed Fisher’s z-transformation to approximately normalize the distribution of the correlation coefficients. The Fisher-transformed correlation matrix was binarized to preserve edge density of 15%. It has been said that a network structure with an edge density of 5% to 50% keeps the small world topology [19,20]. In addition, Bernhardt et al. [21] analyzed a network with 15% edge density as a representative for the functional network structure. The binarized matrix is regarded as the adjacency matrix of the undirected graph, with fNIRS measurement channel as the node and the functional connectivity as the binary edge. Moreover, graph theory analysis was used in this study; the degree, which is one of the well-known network metrics, was calculated using Brain Connectivity Toolbox (https://sites.google.com/site/bctnet/). The degree $k_{i}$ of a certain node *i* (corresponding to the *i*th measurement channel of fNIRS system in this study) is expressed by Equation (1), where *N* is the total number of the nodes in the network and *a* is the element of the adjacency matrix.

(1) $$k_{i}=\sum_{j\in N}a_{ij}$$

$k_{i}$ indicates the number of nodes that are functionally connected with other nodes in the network and reflects the importance of the nodes in the network [14].

#### 2.4.3. Behavioral Data Analysis

Synchronization error (SE) was calculated from the data related to the response time in the task. SE shows the difference between the time durations when the subject pressed the button (response) and the auditory cue was given (stimulus; Equation (2)).

(2) SE(n)=Response(n)-Stimulus(n)

If the subject pressed the button before the cue, SE takes a negative value, and, if they pressed the button after the cue, SE takes a positive value. Here, the SE for the initial signal in the task block was excluded from the analysis because the initial stimulus in the task block was not regarded as the model signal that simulates the others’ tapping, but as a reminding signal for starting of the task. In addition, the average values of SE in each cluster were calculated and compared with each other.

### 2.5. Subject Classification

Since SE varies between participants as much as between behaviors, it is not necessarily a good behavioral measure of the two strategies we assumed. Here, we used data-driven approach to separate the participants into some groups by data clustering in terms of the similarity of the brain network, and compared the network measure and the SE among groups. Ward’s method, which is a well-known hierarchical clustering method, was used to categorize the subjects into multiple groups.

## 3. Results

### 3.1. Spatial Registration of the Measurement Channels to the Brain Regions

The measurement channel of each subject was aligned to the MNI space. Each measurement channel was associated with the highest percentage of the brain regions among the estimated brain regions of each subject. Table 1 shows the brain regions corresponding to each channel.

### 3.2. Subject Classification

**Figure 4:**
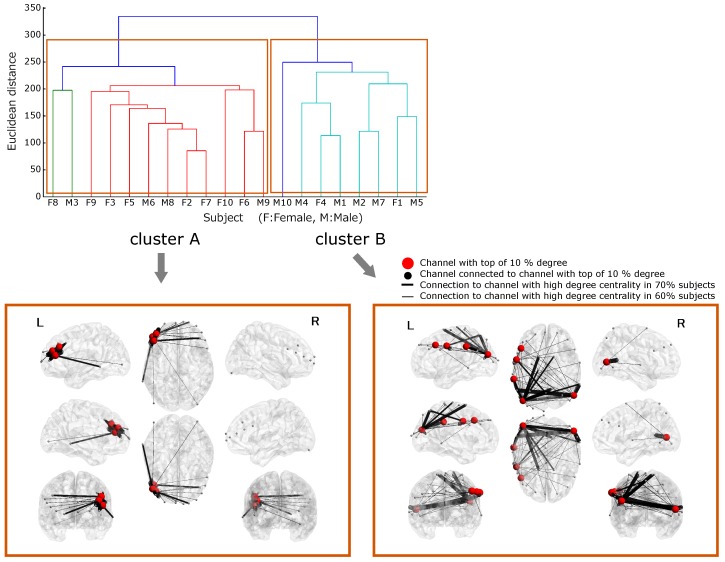
Brain states were classified by hierarchical clustering. Two clusters with the greatest distance were revealed. The red circle indicates the central area of the network with a high degree. Cluster A was in the frontal lobe, and Cluster B was in the temporal and lateral lobes. Letters L and R indicate the left and right sides of the brain, respectively.

Figure 4 shows the dendrogram obtained as a result of the hierarchical clustering. The vertical axis shows the distance between the clusters, and the horizontal axis shows the subjects. Based on this result, the subjects were classified into groups with the largest distance between the clusters. It revealed that there were two brain states during the synchronized tapping task. We compared the two groups based on their brain states, and the behavioral data of each cluster.

### 3.3. Functional Connectivity in Cluster A

The regions with top 10% degree comprised the left middle frontal gyrus (LMFG), and the left triangular part of inferior frontal gyrus (LTrIFG). Figure 4 shows the network connections around the LMFG and LTrIFG, found in 60% of the subjects. It was drawn using BrainNetViewer 1.53 (https://www.nitrc.org/projects/bnv/). These two regions were connected to each other. In addition, LMFG was connected with the right middle frontal gyrus (RMFG), and the right superior frontal gyrus (RSFG). Furthermore, unpaired two-sample *t*-test for the difference between two groups, setting LMFG and LTriIFG as the region-of-interests was conducted. Degree centralities of two regions in Cluster A were significantly higher than those in Cluster B (p<0.05).

### 3.4. Functional Connectivity in Cluster B

The regions with top 10% degree comprised the LMFG, the left middle occipital gyrus (LMOG), the left postcentral gyrus (LPoG), the left supramarginal gyrus (LSMG), and the right middle temporal gyrus (RMTG). In addition to Cluster A, Figure 4 shows the functional connections between these regions and the other regions. Regions with the top 10% degree were interconnected, and all the top 10%-degree regions had at least one connection with each other. LMFG had a connection with LPoG and LMOG, which are regions within the top 10% degree. In addition, LMOG had connections with RMTG, LSMG, precuneus, and the right superior temporal gyrus (RSTG). Moreover, unpaired two-sample *t*-test for the difference between two groups, setting LMFG, LMOG, LPoG, LSMG and RMTG as the region-of-interests was conducted. The results of the *t*-test indicated that the degree centralities of the LMOG and LPoG in Cluster B were significantly higher than those in Cluster A (p<0.05).

### 3.5. Analysis of Behavioral Data

We used SE values for the analysis of behavioral data. The average values of SE in Cluster A, and Cluster B were 90.0 ± 154.9 ms and −8.9 ± 261.5 ms, respectively. There was no significant difference between the two clusters (p<0.05).

## 4. Discussion

The top 10%-degree brain region indicate that they are extensively connected to other regions of the brain and are therefore high centrality regions in the functional network. As a result of clustering on the basis of degree, we grouped the subjects into two clusters, Cluster A with network centrality in the frontal lobe and Cluster B with network centrality in the temporal and lateral lobes, during timing synchronization. In addition, at least one of the central regions in both clusters had a mutual connection. This suggests that the principal network in timing synchronization consists only of the central regions of the various network clusters, which are interconnected with each other.

For the subjects in Cluster A, LMFG and LTrIFG corresponded to the central region of the network, with top 10% degree, and both regions are involved in theory of mind (ToM) [22]. ToM is one of the social skills necessary to understand the mind of the others, and also the intention behind their action. IFG is also involved in mirror neuron systems [23]. The mirror neuron system is a neural mechanism that allows unconscious understanding of the behavior and intention of the others; this neural basis is also found in infants [24]. In addition, MFG has been reported to be involved in sustaining attention [25]. Thus, by functionally connecting the region related to observation and imitation of behavior, and the MFG which sustains attention, the subjects were able to synchronize their response time. They observed and responded to the sound stimulus, and modeled the others’ behavior while paying attention to the next cue. Furthermore, the mean values of the SE in Cluster A shows the subjects’ tendency to respond slowly to the cues. This suggests that the subjects responded “reactively” to the cue. Since imitating the others indicates reacting to the opponent’s behavior, the result of this SE value supports the results of the brain function network (Cluster A).

On the other hand, LMFG, LPoG, LMOG, LSMG, and RMTG were the regions with the top 10% degree in subjects of Cluster B, and these regions corresponded to the central region of the network. LMFG associated with sustenance of attention had many connections with the other high degree regions. This indicates that the highest degree of attention was paid on the functions of the other regions. LPoG is a somatosensory cortex, and we think that it became the center of the network because of its relation with the urge to push the button [26]. LMOG is a visual cortex. In this experiment, fixation point was always presented as a visual stimulus. Therefore, it is conceivable that factors, which are important in the network, do not include processing of visual information. Summerfield et al. [27] reported that this region is activated in the expectation of the next stimulus. In addition, it is reported that LSMG, which is connected to LMOG and forms the center of the network, becomes active when responding to predictable stimuli [28]. STS, including RSTG and RMTG, and precuneus are regions related to the ToM and are thought to be involved in understanding the intention of the cue [22]. Taken together, we conclude that the subjects were synchronizing their response time with the stimulus using LMOG, which is activated in anticipation of the next cue, and is also connected to the regions involved in understanding the meaning of the cues such as RSTG, RMTG, and precuneus. In addition, subjects tended to respond before the cues were generated, as demonstrated by the mean values of SE in Cluster B. Based on these results, we conclude that the subjects in Cluster B proactively responded to the stimulus.

The results of Clusters A and B suggest that timing coordination involved both imitative and predictive behaviors. In previous studies, the mirror neuronal systems have been shown to be involved in recognition of the others, and it has been clarified that humans understand the behavior of the others by simulating them in their interbrain or actual behavior [24]. Here, as shown by the Cluster A network, the mirror neurons are involved in this task along with the imitative behavior to coordinate with the others to synchronize timing. In addition to imitation, as shown by the network result of Cluster B, a network involved in anticipation was formed, suggesting the need to predict the timing of others.

These results reveal the central region and the structural features of the brain functional network associated with time coordination. The participants were asked to synchronize with the presence of the sound stimuli, and the stimuli were regarded as the opponent’s behavior in this study. However, the inter-stimulus interval did not differ in response to the subjects’ tapping. Therefore, further studies are required to confirm whether the same result can be obtained when the timing is synchronized as the two persons mutually adapt.

One of the limitations of the current study is the motion artifact (MA) correction method. It is a crucial issue in fNIRS-based analysis, and many methods have been proposed recently, including principle component analysis, spline interpolation, Kalman filtering, wavelet filtering and correlation-based signal improvement [29,30]. However, accuracy of MA correction significantly differs among different methods and the methodological differences lead to different statistical results [31].

## 5. Conclusions

Joint action can be regarded as social interactions in which more than two people coordinate their behavior to change the environment. The ability to coordinate our behavior with external stimulus is essential to achieve a joint action. In this study, using synchronous tapping task, brain activity was measured by fNIRS when people coordinated their timing. In addition, brain functional networks in the task were examined using the graph theory analysis. We suggest that there are two kinds of brain function networks related to the task being performed. We also showed that these networks comprise a major network. Furthermore, from the point of view of the two networks and behaviors, it was revealed that both expectative behavior and imitative behavior are involved in time synchronization. Our findings demonstrate that it is possible to take actions that are adapted to other people’s actions by predicting and imitating behaviors that have been regarded as important in social interactions.

## Figures and Tables

**Table 1:** The associated brain region corresponding to all the channels (116 channels). Each measurement channel was associated with the highest percentage of the brain regions for each subject.

Channel	Region		Channel	Region
1	right postcentral gyrus		59	left middle occipital gyrus
2	right precentral gyrus		60	left middle occipital gyrus
3	right middle frontal gyrus		61	left middle occipital gyrus
4	right superior frontal gyrus		62	left calcarine sulcus
5	right medial superior frontal gyrus		63	right inferior occipital gyrus
6	left superior frontal gyrus		64	right middle occipital gyrus
7	left middle frontal gyrus		65	right middle temporal gyrus
8	left middle frontal gyrus		66	right superior temporal gyrus
9	left postcentral gyrus		67	left middle temporal gyrus
10	right supramarginal gyrus		68	left middle temporal gyrus
11	right precentral gyrus		69	left inferior occipital gyrus
12	right triangular part of inferior frontal gyrus		70	left lingual gyrus
13	right middle frontal gyrus		71	left cerebellum crus II
14	right superior frontal gyrus		72	right lingual gyrus
15	left medial superior frontal gyrus		73	right inferior occipital gyrus
16	left middle frontal gyrus		74	right inferior temporal gyrus
17	left middle frontal gyrus		75	right middle temporal gyrus
18	left precentral gyrus		76	left middle temporal gyrus
19	left postcentral gyrus		77	left middle temporal gyrus
20	right postcentral gyrus		78	left cerebellum crus I
21	right triangular part of inferior frontal gyrus		79	left cerebellum crus I
22	right middle frontal gyrus		80	left cerebellum crus II
23	right middle frontal gyrus		81	right lingual gyrus
24	right medial superior frontal gyrus		82	right cerebellum crus I
25	left superior frontal gyrus		83	right cerebellum crus I
26	left middle frontal gyrus		84	right inferior temporal gyrus
27	left triangular part of inferior frontal gyrus		85	right middle temporal gyrus
28	left postcentral gyrus		86	left inferior temporal gyrus
29	right superior temporal gyrus		87	left cerebellum crus I
30	right opercular part of inferior frontal gyrus		88	left cerebellum crus II
31	right triangular part of inferior frontal gyrus		89	left cerebellum crus III
32	right middle frontal gyrus		90	left cerebellum crus IV
33	right superior frontal gyrus		91	right cerebellum crus II
34	left medial superior frontal gyrus		92	right cerebellum crus II
35	left middle frontal gyrus		93	right cerebellum crus I
36	left triangular part of inferior frontal gyrus		94	right inferior temporal gyrus
37	left triangular part of inferior frontal gyrus		95	right superior frontal gyrus
38	left postcentral gyrus		96	right precentral gyrus
39	right middle temporal gyrus		97	right superior parietal cortex
40	right triangular part of inferior frontal gyrus		98	right superior parietal cortex
41	right middle frontal gyrus		99	right superior frontal gyrus
42	right superior frontal gyrus		100	right superior frontal gyrus
43	right medial superior frontal gyrus		101	right precentral gyrus
44	left superior frontal gyrus		102	right superior parietal cortex
45	left middle frontal gyrus		103	right cuneus
46	left triangular part of inferior frontal gyrus		104	supplementary motor area
47	left superior temporal gyrus		105	left paracentral lobule
48	left supramarginal gyrus		106	left precuneus
49	left middle temporal gyrus		107	left precuneus
50	left middle occipital gyrus		108	left superior frontal gyrus
51	left middle occipital gyrus		109	left superior frontal gyrus
52	left calcarine sulcus		110	left paracentral lobule
53	right superior occipital gyrus		111	left superior parietal cortex
54	right middle occipital gyrus		112	left superior parietal cortex
55	right middle temporal gyrus		113	left superior frontal gyrus
56	right superior temporal gyrus		114	left precentral gyrus
57	right supramarginal gyrus		115	left postcentral gyrus
58	left middle temporal gyrus		116	left superior parietal cortex

## Abbreviations

The following abbreviations are used in this manuscript:

fNIRS	functional near-infrared spectroscopy
fMRI	functional magnetic resonance imaging
MNI	Montreal Neurological Institute
POTATo	platform for optical topography analysis tools
Hb	hemoglobin
SE	synchronization error
LMFG	left middle frontal gyrus
TrIFG	triangular part of inferior frontal gyrus
RMFG	right middle frontal gyrus
RSFG	right superior frontal gyrus
LMOG	left middle occipital gyrus
LPoG	left postcentral gyrus
LSMG	left supramarginal gyrus
RMTG	right middle temporal gyrus
RSFG	right superior temporal gyrus
ToM	theory of mind

## References

[1] Wiltermuth S.S., Heath C. (2009). Synchrony and cooperation. Psychol. Sci..

[2] Rabinowitch T.C., Knafo-Noam A. (2015). Synchronous rhythmic interaction enhances children’s perceived similarity and closeness towards each other. PLoS ONE.

[3] Sebanz N., Bekkering H., Knoblich G. (2006). Joint action: bodies and minds moving together. Trends Cogn. Sci..

[4] Repp B.H. (2005). Sensorimotor synchronization: A review of the tapping literature. Psychon. Bull. Rev..

[5] Thaut M.H. (2003). Neural basis of rhythmic timing networks in the human brain. Ann. N. Y. Acad. Sci..

[6] Chen J.L., Zatorre R.J., Penhune V.B. (2006). Interactions between auditory and dorsal premotor cortex during synchronization to musical rhythms. Neuroimage.

[7] Lewis P.A., Wing A., Pope P., Praamstra P., Miall R. (2004). Brain activity correlates differentially with increasing temporal complexity of rhythms during initialisation, synchronisation, and continuation phases of paced finger tapping. Neuropsychologia.

[8] Mayville J.M., Jantzen K.J., Fuchs A., Steinberg F.L., Kelso J.S. (2002). Cortical and subcortical networks underlying syncopated and synchronized coordination revealed using fMRI. Hum. Brain Map..

[9] Irani F., Platek S.M., Bunce S., Ruocco A.C., Chute D. (2007). Functional near infrared spectroscopy (fNIRS): an emerging neuroimaging technology with important applications for the study of brain disorders. Clin. Neuropsychol..

[10] Minagawa-Kawai Y., Matsuoka S., Dan I., Naoi N., Nakamura K., Kojima S. (2008). Prefrontal activation associated with social attachment: Facial-emotion recognition in mothers and infants. Cereb. Cortex.

[11] Sanz-Arigita E.J., Schoonheim M.M., Damoiseaux J.S., Rombouts S.A., Maris E., Barkhof F., Scheltens P., Stam C.J. (2010). Loss of ‘small-world’networks in Alzheimer’s disease: graph analysis of FMRI resting-state functional connectivity. PLoS ONE.

[12] Sporns O., Tononi G., Kötter R. (2005). The human connectome: A structural description of the human brain. PLoS Comput. Biol..

[13] Van Dijk K.R., Sabuncu M.R., Buckner R.L. (2012). The influence of head motion on intrinsic functional connectivity MRI. Neuroimage.

[14] Rubinov M., Sporns O. (2010). Complex network measures of brain connectivity: uses and interpretations. Neuroimage.

[15] Maki A., Yamashita Y., Ito Y., Watanabe E., Mayanagi Y., Koizumi H. (1995). Spatial and temporal analysis of human motor activity using noninvasive NIR topography. Med. Phys..

[16] Niu H., Lu C.M., Zhu C., Khadka S., Tian F., Lin Z.J., Liu H. (2011). Resting-state functional connectivity assessed with two diffuse optical tomographic systems. J. Biomed. Opt..

[17] Imai M., Watanabe H., Yasui K., Kimura Y., Shitara Y., Tsuchida S., Takahashi N., Taga G. (2014). Functional connectivity of the cortex of term and preterm infants and infants with Down’s syndrome. Neuroimage.

[18] Pena M., Maki A., Kovačić D., Dehaene-Lambertz G., Koizumi H., Bouquet F., Mehler J. (2003). Sounds and silence: An optical topography study of language recognition at birth. Proc. Natl. Acad. Sci. USA.

[19] Achard S., Bullmore E. (2007). Efficiency and cost of economical brain functional networks. PLoS Comput. Biol..

[20] Wang J., Wang L., Zang Y., Yang H., Tang H., Gong Q., Chen Z., Zhu C., He Y. (2009). Parcellation-dependent small-world brain functional networks: a resting-state fMRI study. Hum. Brain Map..

[21] Bernhardt B.C., Chen Z., He Y., Evans A.C., Bernasconi N. (2011). Graph-theoretical analysis reveals disrupted small-world organization of cortical thickness correlation networks in temporal lobe epilepsy. Cereb. Cortex.

[22] Powell J.L., Grossi D., Corcoran R., Gobet F., Garcia-Finana M. (2017). The neural correlates of theory of mind and their role during empathy and the game of chess: A functional magnetic resonance imaging study. Neuroscience.

[23] Kilner J.M., Neal A., Weiskopf N., Friston K.J., Frith C.D. (2009). Evidence of mirror neurons in human inferior frontal gyrus. J. Neurosci..

[24] Dapretto M., Davies M.S., Pfeifer J.H., Scott A.A., Sigman M., Bookheimer S.Y., Iacoboni M. (2006). Understanding emotions in others: Mirror neuron dysfunction in children with autism spectrum disorders. Nat. Neurosci..

[25] Andersson M., Ystad M., Lundervold A., Lundervold A.J. (2009). Correlations between measures of executive attention and cortical thickness of left posterior middle frontal gyrus-a dichotic listening study. Behav. Brain Funct..

[26] Yan B., Li K., Xu J., Wang W., Li K., Liu H., Shan B., Tang X. (2005). Acupoint-specific fMRI patterns in human brain. Neurosci. Lett..

[27] Summerfield C., Koechlin E. (2008). A neural representation of prior information during perceptual inference. Neuron.

[28] Coull J., Nobre A. (2008). Dissociating explicit timing from temporal expectation with fMRI. Curr. Opin. Neurobiol..

[29] Brigadoi S., Ceccherini L., Cutini S., Scarpa F., Scatturin P., Selb J., Gagnon L., Boas D.A., Cooper R.J. (2014). Motion artifacts in functional near-infrared spectroscopy: A comparison of motion correction techniques applied to real cognitive data. NeuroImage.

[30] Chiarelli A.M., Maclin E.L., Fabiani M., Gratton G. (2015). A kurtosis-based wavelet algorithm for motion artifact correction of fNIRS data. NeuroImage.

[31] Pfeifer M.D., Scholkmann F., Labruyère R. (2018). Signal Processing in Functional Near-Infrared Spectroscopy (fNIRS): Methodological Differences Lead to Different Statistical Results. Front. Hum. Neurosci..

